# Development of a machine vision-based weight prediction system of butterhead lettuce (*Lactuca sativa* L.) using deep learning models for industrial plant factory

**DOI:** 10.3389/fpls.2024.1365266

**Published:** 2024-06-05

**Authors:** Jung-Sun Gloria Kim, Seongje Moon, Junyoung Park, Taehyeong Kim, Soo Chung

**Affiliations:** ^1^ Department of Biosystems Engineering, Seoul National University, Seoul, Republic of Korea; ^2^ Integrated Major in Global Smart Farm, Seoul National University, Seoul, Republic of Korea; ^3^ Research Institute of Agriculture and Life Sciences, Seoul National University, Seoul, Republic of Korea

**Keywords:** controlled-environment agriculture, convolutional neural networks, commercialized plant factory, computer vision, data acquisition system, indoor farming, linear motion guide, regression model

## Abstract

**Introduction:**

Indoor agriculture, especially plant factories, becomes essential because of the advantages of cultivating crops yearly to address global food shortages. Plant factories have been growing in scale as commercialized. Developing an on-site system that estimates the fresh weight of crops non-destructively for decision-making on harvest time is necessary to maximize yield and profits. However, a multi-layer growing environment with on-site workers is too confined and crowded to develop a high-performance system.

This research developed a machine vision-based fresh weight estimation system to monitor crops from the transplant stage to harvest with less physical labor in an on-site industrial plant factory.

**Methods:**

A linear motion guide with a camera rail moving in both the x-axis and y-axis directions was produced and mounted on a cultivating rack with a height under 35 cm to get consistent images of crops from the top view. Raspberry Pi4 controlled its operation to capture images automatically every hour. The fresh weight was manually measured eleven times for four months to use as the ground-truth weight of the models. The attained images were preprocessed and used to develop weight prediction models based on manual and automatic feature extraction.

**Results and discussion:**

The performance of models was compared, and the best performance among them was the automatic feature extraction-based model using convolutional neural networks (CNN; ResNet18). The CNN-based model on automatic feature extraction from images performed much better than any other manual feature extraction-based models with 0.95 of the coefficients of determination (R^2^) and 8.06 g of root mean square error (RMSE). However, another multiplayer perceptron model (MLP_2) was more appropriate to be adopted on-site since it showed around nine times faster inference time than CNN with a little less R^2^ (0.93). Through this study, field workers in a confined indoor farming environment can measure the fresh weight of crops non-destructively and easily. In addition, it would help to decide when to harvest on the spot.

## 1 Introduction

Food security has been seriously threatened by the global pandemic and geopolitical tensions such as COVID-19 and the Russia–Ukraine war (Farcas et al., 2020; Ben Hassen and El Bilali, 2022), along with overpopulation, lower arable lands, and climate change (Hati and Singh, 2021). Overpopulation is anticipated to reach 9.7 billion in 2050, and the demand for food will increase by 70% from the current levels (World Population Prospects - Population Division - United Nations, 2022). In addition, arable land has been decreasing while the urban landscape has expanded (Brain et al., 2023). Climate change, has worsened and has become a reality. According to the European Commission’s Copernicus Climate Change Service, July 2023 was the hottest month in Europe because records experienced severe drought, which has significantly impacted agricultural yields. Therefore, improving food production has become one of the most critical issues in the world, and investments in related systems have risen, requiring commercialization (Adenäuer et al., 2023). In this context, several studies are expected to improve crop production systems.

Controlled-environment agriculture (CEA), such as plant factories, has become one of the most representative ways to improve the efficiency of crop production because it allows growers to use less cultivated land and cultivate crops year-round while minimizing damage from diseases and insects (Kozai and Sasaki, 2013; Mitchell, 2022). This can lead to increased food access nationwide, strengthening food security against the global problems mentioned above. Currently, plant factories with state-of-the-art technology, such as precision farming, are drawing public attention, investment, and development (Hati and Singh, 2021; Chamara et al., 2022). It allows cultivators to collect information on the growth state of crops, such as morphological features and weight in real-time, and automatically controls the environment, resulting in maximum yields and quality (Reyes-Yanes et al., 2020).

Among growth information, the fresh weight of crops has been considered an important indicator for monitoring plant growth to increase productivity and profitability (Mokhtar et al., 2022; Gang et al., 2022a; Tong et al., 2023). This indicates that plant growth rate and uniformity play a significant role in monitoring harvest time and the occurrence of disease (Zhang et al., 2022). Notably, plant factories must track the fresh weight of crops because they are cultured in bulk in the system. This is because the owner should meet the ordered quantity and quality, including certain sizes and weights, and have a specific date of shipping when an indoor farm is commercialized (Petropoulou et al., 2023). A delay in shipment is one of the most severe problems in commercial indoor farming. From economic and industrial viewpoints, a delay in shipment increases operation and maintenance costs and decreases product quality, undoubtedly leading to a decline in profits. Therefore, an automatic weight estimation system is required to determine the exact harvest time and to reduce unnecessary expenses.

Normally, most weighing processes are performed only after harvesting. For continuous monitoring, fresh weight could not be measured during cultivation. In addition, workers often carry the crops to an existing weighing system, such as a hanging load cell, electronic, or floor scale, to measure their fresh weight. This requires a lot of labor, working hours, and increased costs. Direct contact with crops is inevitable, increasing the stress of crops and leading to growth inhibition. Therefore, an automatic and nondestructive weighing system is necessary.

Recently, machine vision has been implemented for weight prediction because of its noninvasive and nonintrusive characteristics to demonstrate a contactless weighing system (Hati and Singh, 2021; Lou et al., 2022; Ojo and Zahid, 2023). Most previous studies collected images by phone, which caused considerable confusion when developing prediction models (Moon et al., 2020). In addition, there was a case where only a small amount of data could be obtained despite using multiple cameras because the locations of the cameras were fixed and had limited coverage of view (Lee, 2008). They have also been implemented in experimental greenhouses and laboratories, controlling most of the environment (Jiang et al., 2018; Reyes-Yanes et al., 2020). Many researchers have used public data, such as datasets collected from the lettuce planting laboratory at Wageningen University and research in the Netherlands, to develop machine-vision-based models (Lin et al., 2022; Zhang et al., 2022; Gang et al., 2022a). However, a large number of consistent quality images of crops are necessary to develop a fresh weight prediction model using images collected by the machine vision system. The best approach is to set up an on-site data acquisition system to optimize the actual users of the system.

Actual industrial plant factories operate under different conditions, and it is difficult to obtain images in any case. In greenhouses and laboratories, the air space above crops is much larger than that in plant factories, because most systems do not use vertically stacked growing beds. Therefore, cameras can be fixed on the top above crops with a height of over 1 m (Lee, 2008; Ojo and Zahid, 2023), whereas it is difficult to apply in plant factories that require a limited height of less than 50 cm. The limited height increases the difficulty of installing an image-acquisition system. Furthermore, there are differences between experimental and industrial plant factories. Experimental locations can be modified in systems as intended so that they have only a few hindrances in the experimental process (Chen et al., 2016). However, industrial plant factories had to consider the high humidity and sloshed water generated when workers cleaned the floors and cultivating beds during the experiment. In addition, the machine vision system had to occupy minimal space and not interrupt the workflow of workers. Therefore, this study demonstrates an appropriate machine vision system that acquires images under harsh conditions such as narrow, crowded, and high humidity.

Another important factor to consider in industrial factories is that economic and commercial crops should be prudently chosen to enhance the profitability of commercialized plant factories. Lettuce is a relatively fast-growing and commonly produced crop worldwide in plant factories with artificial lighting (PFALs) (Kozai et al., 2019). Lettuce has several varieties, including iceberg, red leaf lettuce, and Latin lettuce. According to the report ‘Hydroponic lettuce market is thriving worldwide during the forecast period 2023–2030,’ butterhead lettuce had the highest market share as of 2022 among the hydroponic lettuce. In this study, butterhead lettuce (*Lactuca sativa* var. capitata L. nidus tenerrima) has been selected since it is one of the most famous crops cultivated in commercialized plant factories such as Aerofarms from New Jersey in the United States of America, Vertical Roots from Edmonton in Canada and PlanTfarm from Pyeongtaek in South Korea consumed a lot as a salad.

This study developed a non-destructive fresh weight prediction system for butterhead lettuce in an industrial plant factory using images collected by an automatic image acquisition system. This machine vision system collects data on crops without contact and with consistency immediately. In addition, we compared manual feature extraction models with various combinations of parameters to automatic feature extraction-based models to determine the best fresh weight prediction model. This on-site data-based model is expected to be better utilized in the field, and it is expected to help estimate the exact fresh weight state of the crop in real time and when to harvest.

## 2 Materials and methods

### 2.1 Field experiment site and target crop

The experiment was conducted inside a T-Farm2 (PlanTFarm Co., Ltd., Gyeonggi-do, Republic of Korea). Air temperature, relative humidity, and carbon dioxide concentration were maintained at 21.2 ± 3°C, 75.6 ± 15%, and 827 ppm on average, respectively, which fits the growth of butterhead lettuce since the optimal range of air temperature and air relative humidity for lettuce is 18°C–25°C and 60%–80%, respectively (Boros et al., 2023). In addition, carbon dioxide stayed in the range of 788 ppm–917 ppm, which is an appropriate concentration for cultivating the lettuce (Zhang et al., 2017). In the case of the nutrient solution, the pH was in the range of 6.29–6.72, with a mean value of 6.46.

**Figure 1:**
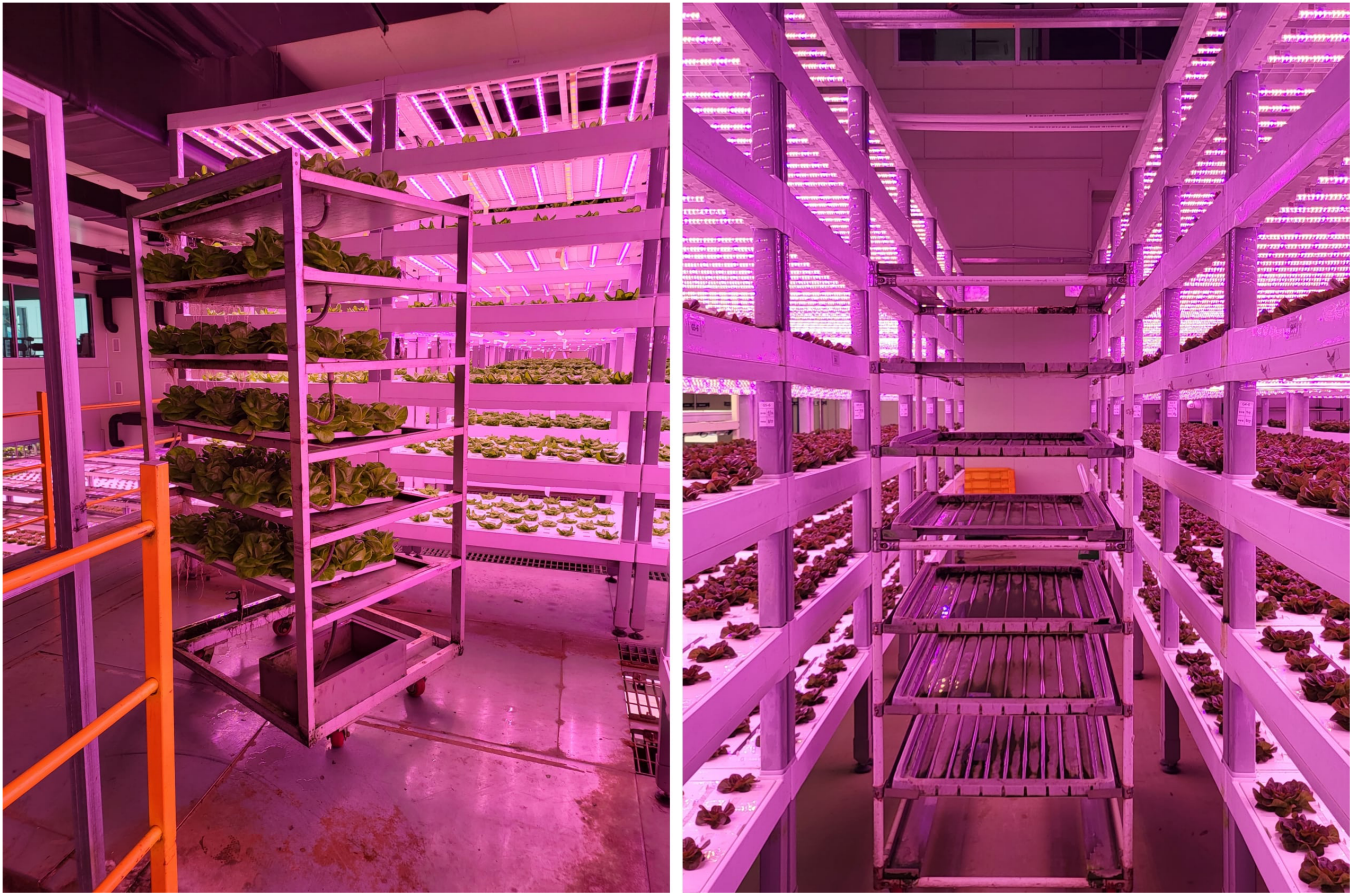
Narrow passages and the height of moving racks for cultivating work in the industrial plant factory.

**Figure 2:**
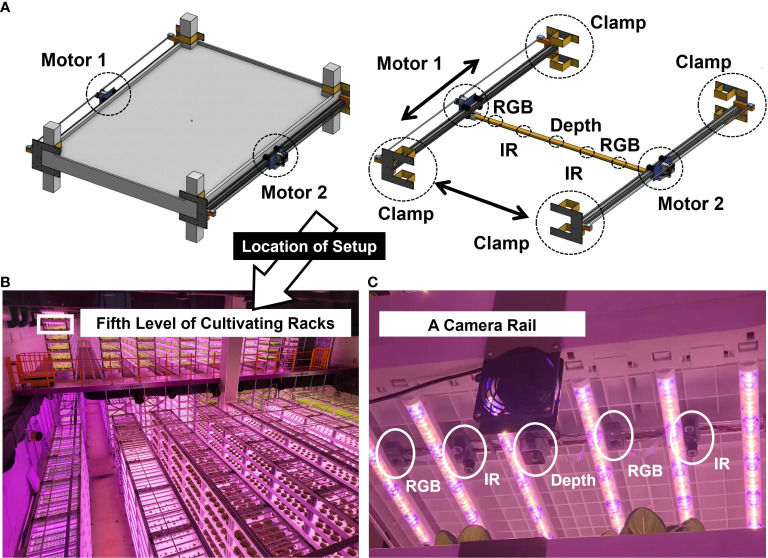
**(A)** A linear motion guide mounted on a cultivating rack and a camera rail with five different cameras (two RGBs, two IRs, and a depth) connected to the linear motion guide; **(B)** the location of the rack installed with the linear motion guide in the industrial plant factory; **(C)** setup of the connected camera rail with five different cameras to the linear motion guide.

A total of 159 Butterhead lettuce (*L, sativa* var. capitata L. nidus tenerrima) was cultivated for 102 days (from 30 September 2022 to 10 January 2023. The location of the cultivation rack of the butterhead lettuce for the experimental application was on the fifth level from the bottom of the cultivation racks to avoid interference from workers during cultivation (**Figures 1**, **2B**).

#### 2.1.1 Image acquisition system

An image acquisition system was designed based on a linear motion guide to attain consistent images automatically and stably on site under harsh environmental conditions, such as high humidity and confined space. The data acquisition system was produced by a company named Robowill from the Republic of Korea.

**Figure 3:**
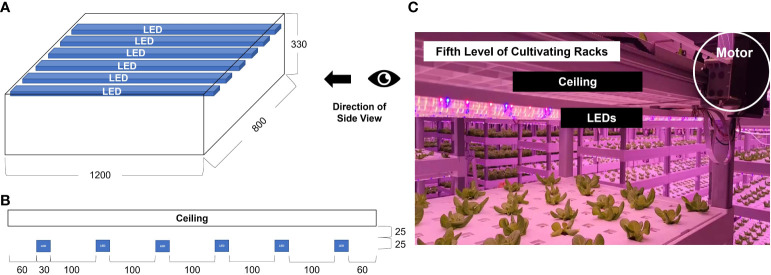
Sketch (unit: mm) of a confined cultivating bed used in the industrial plant factory; **(A)** A three-dimensional front view; **(B)** A side view showing the location of a camera rail, cameras, and LEDs.

An image acquisition system was installed at the top of the cultivation bed. The height from the bottom to the ceiling of the cultivating bed was 330 mm (**Figure 3A**), and the LEDs were installed 25 mm away from the ceiling (**Figure 3B**). The height of the LEDs was 25 mm, and the distance between them was 100 mm. Cameras were installed 50 mm from the ceiling to avoid disturbing the illumination path.

**Figure 4:**
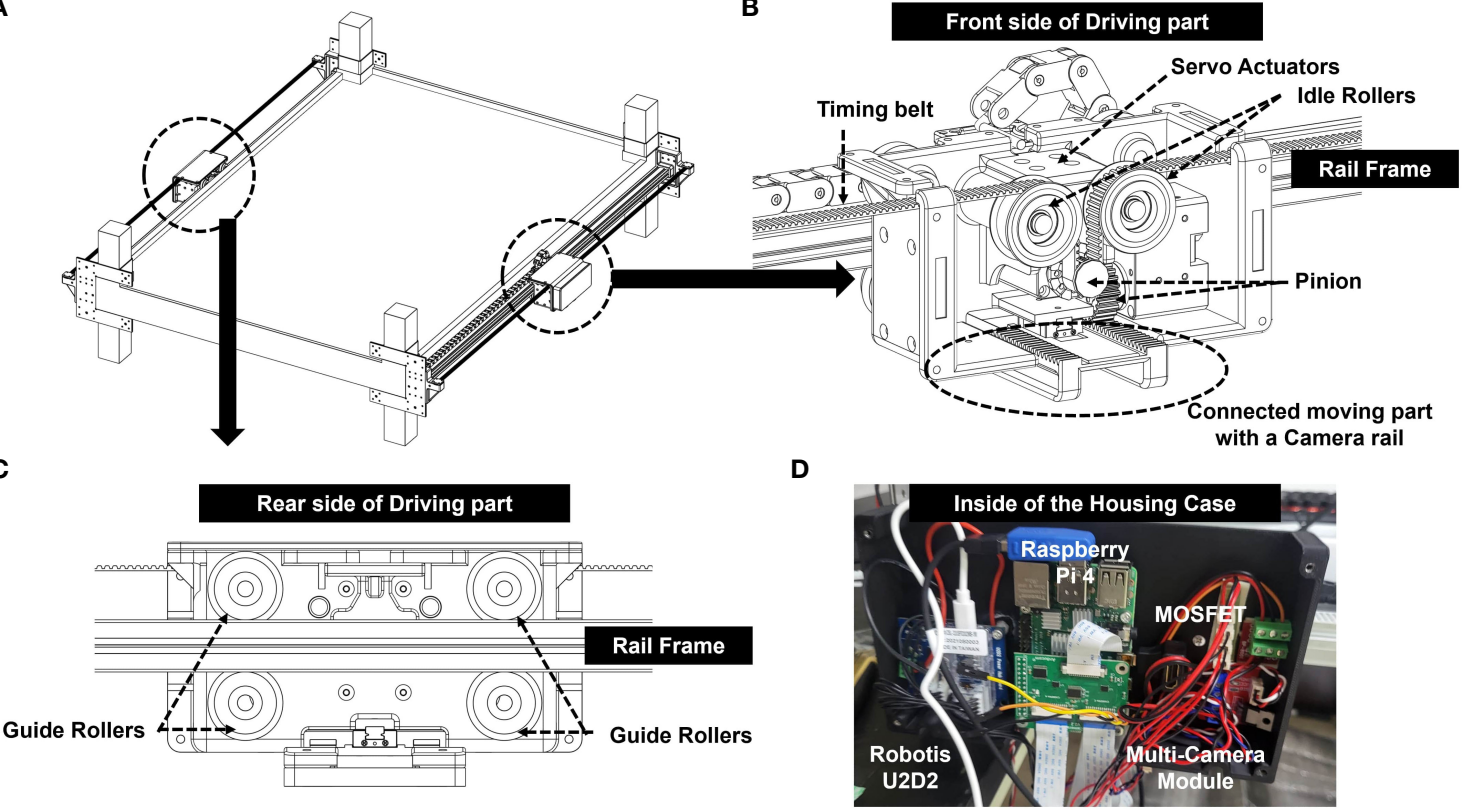
Overall framework and components of the Automatic Image Acquisition System; **(A)** A linear motion guide with two motors; **(B)** Front side of a driving part connected to a rail frame; **(C)** Rear side of a driving part connected to a rail frame; **(D)** Inside of the housing case attached to the outside surface of a driving part.

The present system allowed multiple cameras to be installed within a narrow space, such as the top of a plant factory, by moving the installed terminal rail (**Figure 4B**) connected to cameras in the longitudinal or width direction to capture unstructured data of the crops located below. In addition to taking photos of images, driving parts (**Figure 4A**), such as motors, were safe under the high temperature and humidity of the growing environment. A clamp (**Figure 4A**) fixes the finishing plate provided at the end of the rail frame and is removably attached to the support pillar of the facility.

A pair of rail frames (**Figure 4C**) were arranged along the longitudinal direction to be spaced to a certain degree in the width direction. A terminal rail was equipped with multiple cameras at the bottom and installed in the width direction such that that both ends were placed on the rail frame. The first driving part is provided on one side of the rail frame, and the terminal rail is moved longitudinally (**Supplementary Video 1**). The second driving part was provided on the other side of the rail frame, and the terminal rail was moved in a longitudinal and width-wise direction (**Supplementary Video 2**).

A total of five cameras were connected to the camera rail: two Raspberry Pi camera modules (V2, Raspberry Pi Foundation, Cambridge, UK) taking RGB images with a resolution of 8-megapixel, two Pi NOIR cameras (Raspberry Pi Infrared Camera Module 2 NOIR) modules, taking infrared images with a resolution of 8-megapixel, and one depth camera of RealSense (D405, Intel, CA, USA) (**Figures 2B, C**). The Pi NOIR camera modules were connected to infrared lighting devices (YR-030) to collect images during the night, because the plant factory turned off the light for eight hours every day from 22:00 to 06:00. IR light was controlled by the MOSFET module for automatic switching on and off at the exact time: switch-on at 22:00 and switch-off at 06:00. The linear motion guide was coated with harmless substances (food-grade lubricants, SuperLube) that did not affect the health of the crop and helped smooth the movement and waterproofing of the motion guide.

The image acquisition system was attached to the highest level of cultivating racks nondestructively using clamps (**Figures 2A, B**). Images were taken every hour, automatically moving in the horizontal and vertical directions (**Figure 2C**). For waterproofing, two parallel driving parts were covered in one case (**Figures 4A, B**). The control unit was attached to the front wall of driver. To specifically describe the control unit, Raspberry Pi 4 (Raspberry Pi Foundation, Cambridge, UK) connected to five cameras and a motor was covered with a case (**Figures 4A, D**). The timing belt and timing pulley were used to move vertically, and a rack and pinion frame were applied for horizontal movement (**Figures 4B, C**). A total of 2,040 aluminum profiles were used as the rail frames. A Robotis Dynamixel XM430-W350 actuators were used as a motorizing servo actuator and Robotis U2D2 was used as a Dynamixel for the USB communication module (**Figure 4D**).

A Secure Shell (SSH) was used to remotely control an image acquisition system that does not require a display for on-site applications. In addition, we used Tmux, a terminal multiplexer, to keep the system operating even if the SSH is disconnected due to problems in the plant factory. The image acquisition system saved images on Google Cloud and the local MCU in the PNG format with a pixel resolution of 3,280 × 2,464.

#### 2.1.2 Manual fresh weight measurement

**Figure 5:**
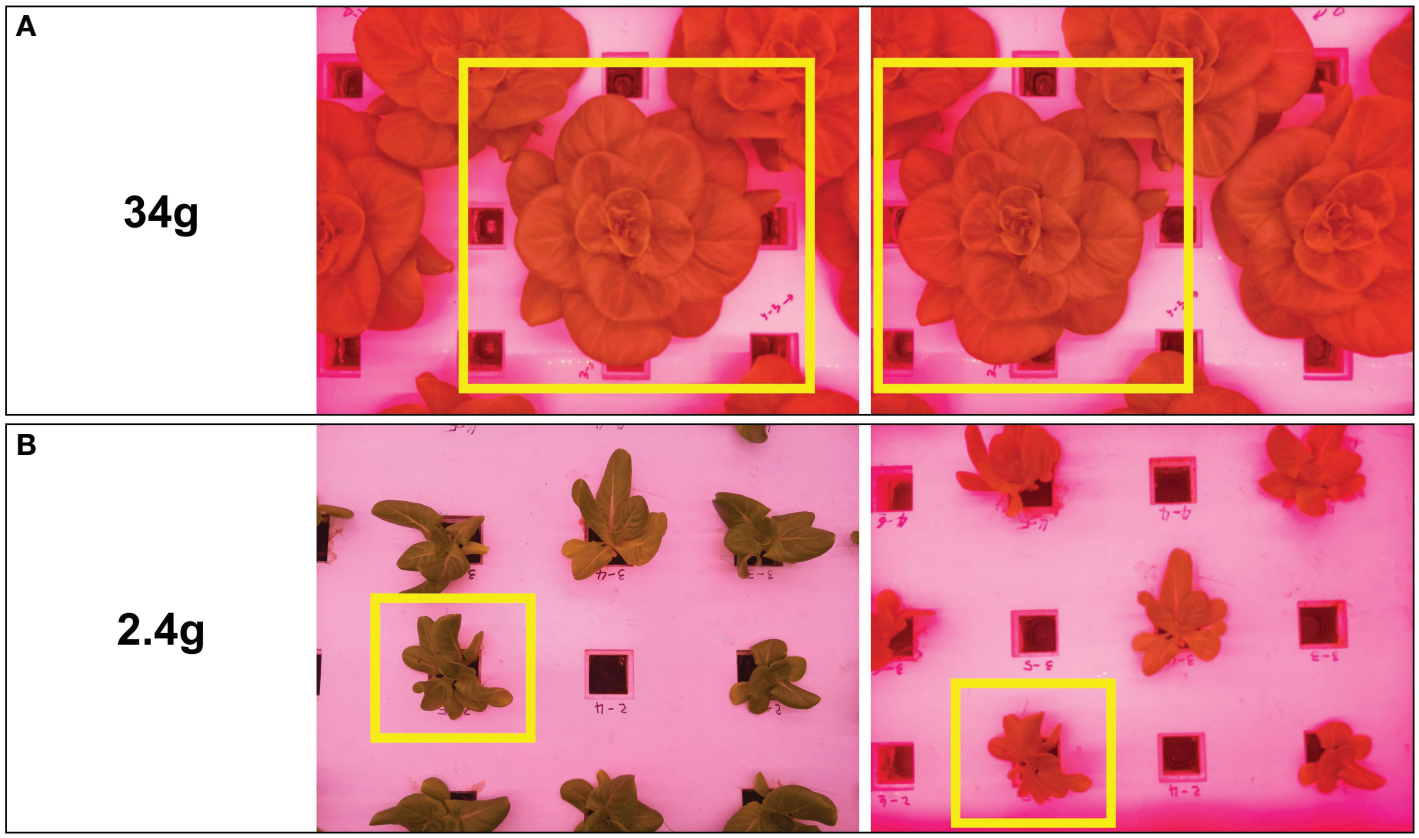
Two different images of the same crop from different locations of cameras with two examples; **(A)** a plant with a fresh weight of 34 g; **(B)** a plant with a fresh weight of 2.4 g.

Images were collected for three months in a row for different stages of lettuce from 23 September 2022 to 10 January 2023, and 159 crops were destructively weighed 11 times (every 5 to 7 days) after removing the root (**Table 1**). Some images with overlapping or partially captured parts were not selected as datasets to develop the models. The number of chosen images matched to fresh weight was 376, as shown in **Table 1**. Images showing the entire part of the crop were selected and used repeatedly, and images showing only parts of the crop were not used because the data acquisition system can be designed to find ways to cover all shapes of each crop at the stage of detection in the future. The linear motion guide automatically moved and captured images at the same height, but at different angles, retaining the position of the top view per crop. Therefore, multiple images can be obtained from different angles for the same crop depending on the location of the cameras. For example, **Figure 5** shows two examples of multiple images of the same plant taken from the location of the cameras. Two plants of 34 g (above images) and 2.4 g (below images) are shown as examples in **Figure 5**. This process produced varied unstructured data for each crop.

**Table 1:** The number of manually measured harvests and the number of corresponding images by date.

Date of Harvest	Days after transplanting (DAT)	Number of Weighed Harvest	Number of Images
30/09/2022	7	12	46
07/10/2022	15	12	45
12/10/2022	0	12	33
21/10/2022	9	12	25
28/10/2022	16	11	66
04/11/2022	23	12	36
04/11/2022	0	12	35
11/11/2022	7	9	9
25/11/2022	21	34	29
27/12/2022	0	9	9
03/01/2023	7	12	23
10/01/2023	14	12	20
Total	–	159	376

### 2.2 Image preprocessing and manual feature extraction

**Figure 6:**
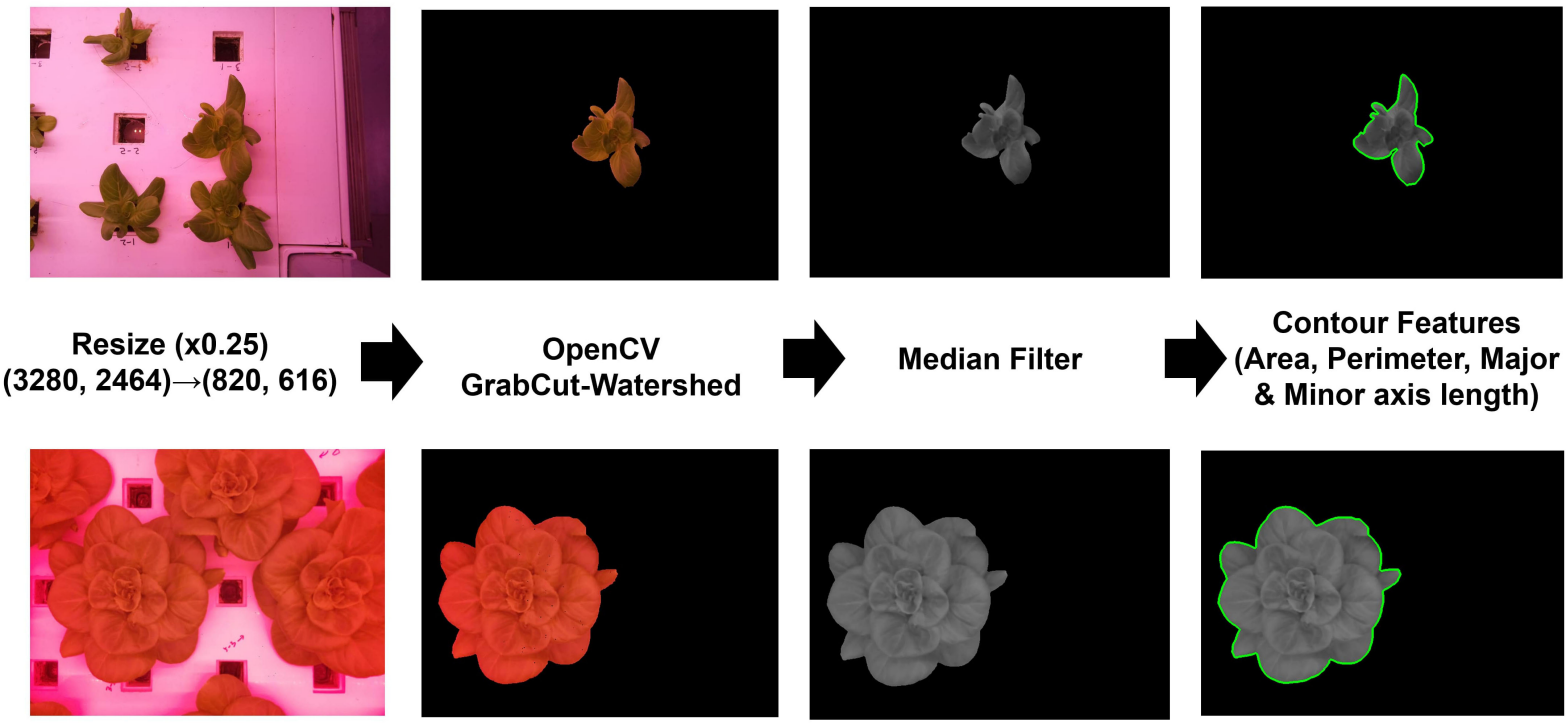
Image preprocessing workflow: Resize, image segmentation, median filtering, and feature extraction.

The collected and chosen images, free from overlapping or partial cut-off issues, were preprocessed using OpenCV 4 with Python to refine the datasets, remove complex backgrounds, and segment one target object from multiple objects in the raw data. All images were resized to 616 × 820 pixels to reduce computational resources. GrabCut, applied with the watershed method and median filter, was used to segment target objects from the background and remove unnoticed noise. This led to a lower failure at the subsequent stage of extracting contour features of the target objects from the images (**Figure 6**). From the contour features, we extracted the values of area (A), perimeter (P), and length of the major axis (MA) and minor axis (MI). A is the area inside the closed curve, and P is the length of the closed curve. MA and MI were extracted by fitting an ellipse; MA was the longest length, and MI was the shortest length of the fitted ellipse. All of these were used as input for fresh weight prediction models.

### 2.3 Fresh weight prediction models

There are two major methods for predicting the fresh weights of crops using computer vision: manual feature extraction (Mortensen et al., 2018) and automatic feature extraction (Lin et al., 2022; Moon et al., 2022a). In other words, manual feature extraction occurs when humans select and extract features manually to predict output values. In contrast, automatic feature extraction occurs when computers select and extract features automatically.

The arranged data for developing the fresh weight estimation models were A, P, MA, MI, images, and fresh weight. The fresh weight values were the responsible (output) variables in the manual and automatic feature-extraction-based models. A, P, MA, and MI were used as explanatory (input) variables in the manual feature-extraction model-based models. For comparison, images were used as explanatory (input) variables in the automatic feature extraction model-based models.

#### 2.3.1 Conventional linear regression models

##### 2.3.1.1 Linear regression based on manual feature extraction

With four manually extracted variables (area, perimeter, major axis length, and minor axis length), linear regression with a single variable (Simple-LR, m=1 in **Equation 2**) and linear regression with multiple variables (Multi-LR) was performed (**Equation 1**). Multiple linear regression models were developed using a combination of two, three, and four independent variables. In total, four simple-LR models and 11 Multi-LR models were developed. All the regression models were developed in Keras.


(1) $$\hat{y}=\beta_{0}+\beta_{1}x_{1}+\beta_{2}x_{2}+\beta_{3}x_{3}+\ldots +\beta_{p}x_{p}+\;\epsilon$$

Where, $\hat{y}$ is the predicted value of the dependent variable, $x_{1}$ to $x_{p}$ are p distinct predictor variables. $\beta_{0}$ represents $\hat{y}$ intercept, and ϵ is the residuals. $\beta_{1}$ to $\beta_{p}$ denote the estimated regression coefficients.

##### 2.3.1.2 Polynomial regression based on manual feature extraction

Polynomial regression is a form of linear regression that fits the nonlinear relationship between the dependent and independent variables. All polynomial regression models were conducted with a second degree (m = 2 in **Equation 2**) and third degree (m = 3 in **Equation 2**) polynomials to form quadratic and cubic expressions, respectively. Simple polynomial regression and multivariate polynomial regression were performed in the same way as above, with a combination of two, three, and four independent variables. In total, eight simple polynomial regression models and twenty-two multiple linear regression models were developed.


(2) $$\hat{y}=\;\beta_{0}+\;\beta_{1}x+\;\beta_{2}x^{2}+\;\beta_{3}x^{3}+\ldots +\;\beta_{m}x^{m}\;+\;\epsilon$$

Where, $\hat{y}$ is the predicted value for the polynomial model with regression coefficients $\beta_{1}$ to $\beta_{m}$ for each degree m and $\hat{y}$ intercept $\beta_{0}$ . It has *m* predictors raised to the power of *I*, where *i* = 1 until *m*. ϵ represents the model’s error term.

#### 2.3.2 Deep learning-based regression models using manual and automatic feature extraction

Several neural networks have been employed to recognize complex nonlinear functions better than traditional statistical regression models (Ong et al., 2008). The architecture of the neural networks is presented in **Table 2**.

**Table 2:** Architectures of deep learning models.

Model	MLP_1	MLP_2	CNN
Input variables	Structured data (A, P, MA, MI)	Unstructured data (Images)	Unstructured data (Images)
Input size	4	784	1 × 28 × 28
Layers	FC-32-ReLU	FC-1568-ReLU	Conv3–64-ReLU
	FC-15-ReLU	FC-392-ReLU	Conv3–128-ReLU
	Dropout(0.5)	Dropout(0.5)	MaxPool(2)
	FC-1-ReLU	FC-1-ReLU	Conv3–256-ReLU
			MaxPool(2)
			Conv3–512-ReLU
			Average Pool(7)
			FC-1
Output size	1 × 1		

FC represents a fully connected layer, a basic form of the neural network, and Conv is a convolution layer. Parameters for Conv are denoted as “{Type of layer}{Kernel size} − {Number of filters} − {Activation function},” and parameters for the other layers are denoted as “{type of layer} − {Number of nodes in the layer} − {Activation function}.” MaxPool represents the maximum pooling.

For the four manually extracted variables as input, multilayer perceptron (MLP_1) had three fully connected linear layers (FC) with different numbers of nodes: 32, 15, and 1 each. The layers with 32 and 15 nodes were followed by the activation function of the rectified linear unit (ReLU). In a fully connected layer with 15 nodes, dropout was employed at a rate of 0.5.

MLP_2 and convolutional neural network (CNN) were applied to unstructured data for automatic feature extraction as regression models. CNN was adopted over other deep learning models because it excels in processing visual data due to its convolutional layers that effectively identify spatial hierarchies and patterns in images. Their architecture was specifically designed to handle the variability and complexity of image data, which is essential for accurate plant weight prediction. Additionally, CNN’s ability to learn features directly from images without manual feature engineering makes them ideal for efficiently analyzing large datasets typically involved in our plant factory conditions. This high performance of CNN has been proven in many previous studies related to weight estimation models (Zhang et al., 2020; Gang et al., 2022a; Moon et al., 2022a). The images were flattened as an input of MLP_2 such that the size of (1 × 28 × 28) was 784. MLP_2 had three fully connected linear layers (FC) with different numbers of nodes: 1,568, 392, and 1. The layers with 1,568 and 392 nodes were followed by an activation function of the rectified linear unit (ReLU). In a fully connected layer with 392 nodes, dropout was employed at a rate of 0.5.

Using Resnet-18, a skip connection is applied to avoid gradient vanishing (He et al., 2016) were used as the CNN model. The convolutional kernels were set to 3 × 3 with a stride of 1, and zero-padding was applied to maintain the same size of output with input size so that edge information could be used. Each layer contained different numbers of feature channels: 64, 128, 256, and 512. The ReLU follows each convolutional layer to ensure training stability. Additionally, maximum pooling was inserted twice after layer of 128-feature channels and 256-feature channels. After the layer of 512-feature channels, global average pool and flattening were performed, followed by a fully connected layer.

## 3 Experiments

### 3.1 Model training

All deep learning models were developed in Pytorch on the Windows Subsystem for Linux (WSL) with a CPU of i9–12900K, GPU of RTX3090 (NVIDIA, Santa Clara, CA, USA), and 64 GB memory.

In the case of automatic feature extraction, the dataset contained samples and labels, and it was split into a training set and a test set with a ratio of 7:3. Of the 376 datasets, 263 were used to develop the training set and 133 were used to develop the test set. In addition, a data loader was constructed with 512 batches to easily access the samples. All fresh weight values were adjusted in scale by multiplying by 0.01 for stability and convergence speed improvement. All input images were adjusted to (28, 28) in size with one channel and were randomly flipped horizontally and vertically.

In the case of manual feature extraction, scaling was performed using MinMax Scaler to ensure that all values existed in the range between 0 and 1 because all values of independent variables had considerable differences in scale. In addition, the Standard Scaler was performed on all response variable values to be standardized, as the distribution’s standard deviation was equal to 1.

MLP_1, MLP_2, and CNN were conducted using the MSE loss function and Adam optimizer with a 0.001 learning rate as the regression models. In the case of MLP_2 and CNN, the number of iterations per epoch was one because the size of the training data was 263 and the batch size was set to 512.

### 3.2 Correlation analysis

The correlation is a statistical measure that expresses the strength of the relationship between two variables. If there are multiple variables, a correlation matrix, a table showing the correlation coefficients between a set of variables, is necessary to find the correlation between all variables. The Pearson’s correlation coefficient (**Equation 3**), known as the correlation coefficient, is a statistical measure of the linear relationship between the two variables. Correlation heatmaps are essential for visualizing the strength of relationships between numerical variables through color coding of the cells. It also allows the identification of outliers and the detection of linear and nonlinear relationships. Correlations between variables were investigated using a heatmap to select the best-fit input variables for fresh weight prediction models.


(3) $$\frac{\sum^{}\left(x_{i}-\;\bar{x}\right)\left(y_{i}-\;\bar{y}\right)\;}{\sqrt{\sum^{}{\left(x_{i}-\;\bar{x}\right)}^{2}\sum {\left(y_{i}-\;\bar{y}\right)}^{2}}}$$

Where *x* and *y* represent two variables, y is the mean value of *x*, and $\bar{y}$ is the mean value of *y*. $x_{i}$ and $y_{i}$ represented different values of *x* and *y*.

Conventional regression models have various compositions of inputs, from one variable to four variables. All combinations of inputs were conducted for a total of 15 cases: (A), (P), (MA), (MI), (A, P), (A, MA), (A, MI), (P, MA), (P, MI), (MA, MI), (A, P, MA), (A, P, MI), (A, MA, MI), (P, MA, MI), and (A, P, MA, MI). MLP_1 was conducted with one set of inputs: (A, P, MA, and MI). MLP_2 and CNN received images as inputs. All models were designed to derive the value of the fresh weight as the output.

### 3.3 Model evaluation metrics

All the developed models were evaluated and compared in terms of model performance with values of the root mean square error (RMSE) and coefficient of determination (R^2^), as shown in **Equation 4** and plots, such as kernel density estimation (KDE), which is used to estimate the underlying probability density function of a dataset (Chen, 2017), allowing to explore the pattern of the data. In addition, the inference time per image (millisecond, ms) was measured because all models were developed for use in industrial plant factories, and the speed of weight estimation can be directly related to the efficiency of the on-site system.


(4) $$\text{RMSE}=\;\frac{\sqrt{\sum_{i=1}^{n}{\left(y_{i}-{\hat{y}}_{i}\right)}^{2}}}{\sqrt{\sum_{i=1}^{n}{\left(y_{i}\right)}^{2}}}$$

$$R^{2}=1-\;\frac{\sum_{i=1}^{n}{\left(y_{i}-{\hat{y}}_{i}\right)}^{2}}{\sum_{i=1}^{n}{\left(y_{i}-{\bar{y}}_{i}\right)}^{2}}$$

where *y* represents the measured value, $\;\hat{y}$ is the predicted value by the models, $\bar{y}$ is the average value of the measured value, and *n* is the number of samples.

## 4 Results and discussion

### 4.1 Correlations between variables

**Figure 7:**
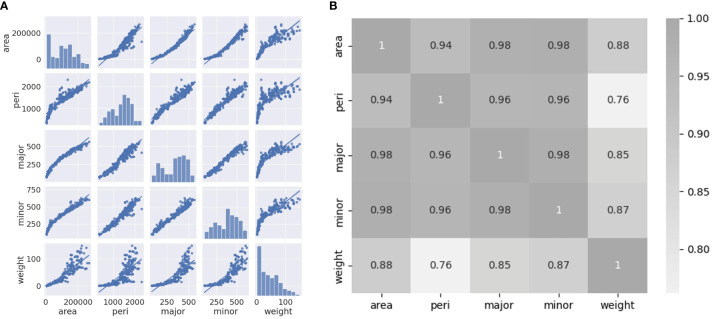
**(A)** Pair plot between variables (area, perimeter, major axis length, minor axis length, and weight); **(B)** Heatmap of Pearson’s correlation coefficients between variables.

Correlation coefficients were analyzed using a pair plot and heatmap to select the appropriate variables. As shown in **Figure 7A**, the relationships between the independent variables and weight are nonlinear. The area, major axis, and minor axis show a high correlation coefficient value to the weight, with a value over 0.85. Moreover, even the lowest correlation coefficient value to the fresh weight was 0.76 (**Figure 7B**). All variables can affect the accuracy of the models when used as input variables.

### 4.2 Performance evaluation and comparison of models

#### 4.2.1 Conventional regression models based on manual feature extraction

The regression model results are shown as the R^2^ and RMSE (g) values in **Tables 3**–**5**. The polynomial regression models performed better than the linear regression models. The Simple-LR (**Table 3**) showed the highest values of 0.77 as R^2^ and 16.21 as RMSE (g) from one variable of A as input, and it had relatively poor performance compared to polynomial regression models with multivariable. Most of the third-degree models showed higher performance (0.90 of R^2^ and 10.63 g of RMSE as best performed model with the combination of A and P, 0.81 of R^2^ and 14.76 g of RMSE on average) than second-degree models (0.88 of R^2^ and 11.87 g of RMSE as best performed model with the combination of A, P, and MA, 0.79 of R^2^ and 15.39 g of RMSE on average). It tends to lower the accuracy of model performances when MI was included with other variables as input, except for one of the results in the Multi-LR models (**Table 4**), which showed the highest values of 0.84 (R^2^) and 13.70 g (RMSE) from three variables: A, P, and MI. Therefore, even if MI is highly correlated with weight (0.87), it may provide redundant information already described in other variables to predict fresh weight and is not essential for fresh weight prediction. However, combinations with P, which showed the lowest correlation value (0.76) with other variables, improved the model performance in all the combinations of other variables, mostly over 0.80 of R^2^, while other combinations without P showed poor model performance, mostly under 0.80 of R^2^ and only P itself as input made the poorest performance, at around 0.6 of R^2^. Therefore, combining P with other variables positively affected the fresh weight prediction by providing more information about crop conditions.

**Table 3:** Results of simple univariate linear and polynomial regression models based on manual feature extraction.

Variables The degree of a polynomial	Area (A)		Perimeter (P)		Major Axis length (MA)		Minor axis length (MI)	
	R^2^	RMSE (g)	R^2^	RMSE (g)	R^2^	RMSE (g)	R^2^	RMSE (g)
1 (Linear)	0.77	16.21	0.60	21.19	0.75	16.77	0.74	17.26
2	0.77	16.34	0.60	21.26	0.77	16.11	0.73	17.48
3	0.83	13.93	0.65	19.93	0.80	15.11	0.76	16.55

Table 4Results of multivariate linear and polynomial regression models based on manual feature extraction.

**Table:** 

Variables The degree of a polynomial	(A, P)		(A, MA)		(A, MI)		(P, MA)	
	R^2^	RMSE (g)	R^2^	RMSE (g)	R^2^	RMSE (g)	R^2^	RMSE (g)
1 (Linear)	0.80	14.86	0.76	16.61	0.77	16.20	0.80	14.87
2	0.86	12.56	0.79	15.53	0.79	15.61	0.83	14.01
3	0.90	10.63	0.80	15.23	0.74	17.24	0.86	12.55

**Table:** 

Variables The degree of a polynomial	(P, MI)		(MA, MI)		(A, P, MA)		(A, P, MI)	
	R^2^	RMSE (g)	R^2^	RMSE (g)	R^2^	RMSE (g)	R^2^	RMSE (g)
1 (Linear)	0.80	15.04	0.73	17.62	0.82	14.16	0.84	13.70
2	0.79	15.66	0.69	18.70	0.88	11.87	0.86	12.65
3	0.83	13.97	0.76	16.65	0.88	11.47	0.85	13.30

**Table:** 

Variables The degree of a polynomial	(A, MA, MI)		(P, MA, MI)		(A, P, MA, MI)			
	R^2^	RMSE (g)	R^2^	RMSE (g)	R^2^	RMSE (g)		
1 (Linear)	0.75	16.97	0.82	14.22	0.83	13.81		
2	0.76	16.66	0.84	13.46	0.85	12.90		
3	0.79	16.44	0.88	11.61	0.88	16.79		

**Table 5:** Results of deep learning models based on manual and automatic feature extraction.

Variables (Features) Model	Manual (A, P, MA, MI)			Automatic from Images		
	R^2^	RMSE (g)	Inference Time per Image (ms)	R^2^	RMSE (g)	Inference Time per Image (ms)
MLP	0.85	14.62	0.002	0.93	9.35	0.003
CNN	–	–		0.95	8.06	0.026

Overall, the best model in performance among conventional regression models was with the combination of A and P as input variables, resulting in 0.90 of R^2^ and 10.63 g of RMSE in the third-degree polynomial regression model. In Simple-LR, A was the best performing variable and P was the poorest-performing variable for the models. However, the model with two variables (A and P) performed best among the conventional regression models. These results show that the variable showing a low correlation value with the target variable should not be excluded when developing models because it can still provide the necessary information for the prediction.

#### 4.2.2 Deep learning regression models based on manual and automatic feature extraction

**Figure 8:**
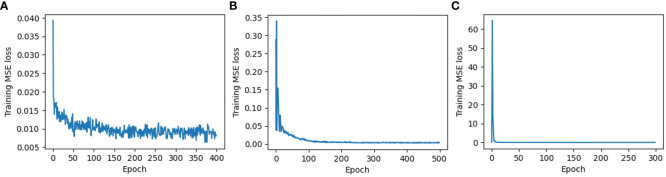
Training MSE loss: **(A)** Multilayer perceptron based on manual feature extraction (MLP_1); **(B)** Multilayer perceptron based on automatic feature extraction (MLP_2); and **(C)** Convolutional neural networks based on automatic feature extraction (CNN).

All the manually extracted features of A, P, MA, and MI were inserted into MLP_1 as input variables, and the R^2^ value of the test set was 0.85 with an RMSE of 14.62 g (**Table 5**) at the epoch of 400 (**Figure 8A**). This was a better performance than simple linear regression models but lower than polynomial regression models, such as the model with the set of A and P as input (0.90 of R^2^ and 10.63 g of RMSE). However, the CNN-applied model with unstructured data based on automatic feature extraction from the input images performed much better. The R^2^ value of the test set was 0.95 with an RMSE of 8.06 g (**Table 5**) at the epoch of 300 (**Figure 8C**), and the ResNet18 model was used as the CNN architecture. This is a much better result than that of previous studies. The research using RGB image-based CNN_284 architecture showed a 0.92 of R^2^ value (Xu et al., 2023). Moreover, it performed similarly to more intricate RGB-D images-based CNN models, which resulted in values of R^2^ around 0.95 (Zhang et al., 2022; Gang et al., 2022a). MLP_2 with images performed relatively well, with an R^2^ of 0.93 and RMSE of 9.35 g, although it showed slightly lower performance than the CNN model. The CNN model is the best, as it shows the highest accuracy of weight estimation. However, MLP_2 is more appropriate for the weight estimation system used in industrial plant factories because of the difference in inference time per image. The speed of the inference time per image is important because it is directly related to the efficiency of the on-site system. If it takes longer than a second to estimate the fresh weight of a plant, the company would be reluctant to use the system because of the slow inference. MLP_2 showed an approximately nine times faster inference time per image than CNN, with a slightly lower accuracy. Specifically, the inference time per image and the R^2^ value of MLP_2 were 0.003 milliseconds (ms) and 0.93, respectively, while those of CNN showed 0.026 ms and 0.95 ms each. Because of the model performance, the input data type is essential for deep-learning models. Automatically extracted features from unstructured data can enhance the performance of deep learning models. In addition, light models that can be run with lower computing power devices, such as a microcontroller unit (MCU), must be used because industrial plant factories have the distinct characteristics of a confined space. Therefore, the on-site fresh-weight estimation model should be simpler. In this aspect, the generated models in this study were appropriate because only RGB and IR images were performed for model development and performed as well as any other complicated models in previous research.

In addition, the number of required epochs for Training MSE loss to converge was 300 in CNN (**Figure 8C**), smaller than MLP_1 (400) (**Figure 8A**) and MLP_2 (500) (**Figure 8B**). The convergence properties of MLP_1 were unstable at the end of the epochs, whereas MLP_2 and CNN exhibited stable convergence properties. Therefore, automatic feature extraction based on images reinforces models by recognizing more complicated interaction functions from the data.

**Figure 9:**
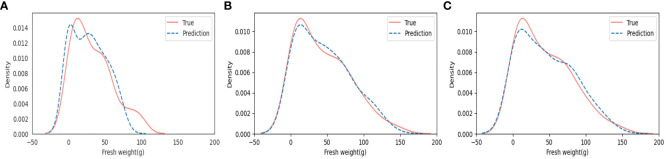
Results of Kernel density estimation **(KDE)** plots: **(A)** Multilayer perceptron based on manual feature extraction (MLP_1); **(B)** Multilayer perceptron based on automatic feature extraction (MLP_2); and **(C)** Convolutional neural networks based on automatic feature extraction (CNN).

We obtained probability density function graphs to estimate the characteristics of the probability distribution from fresh weight and to estimate the values that fresh weight can have and the possible degree of fresh weight on having that value (**Figure 9**). In the results of the KDE plots in **Figure 9**, MLP_1 showed the lowest similarity between true values of manually measured fresh weight (True) and predicted values of fresh weight (Prediction) over the entire range of fresh weight. Meanwhile, both MLP_2 and CNN showed a high similarity between the True and Prediction fresh weights. It is assumed that MLP_2 is more appropriate for use in the range of 0 g to 25 g and 60 g to 100 g than CNN, whereas CNN is more suitable for application in the range of 25 g to 60 g and over 100 g than MLP_2. Different models can be applied to more suitable ranges by considering the model performance. Moreover, it is expected that all weak sections can be strengthened by adding more datasets during the stage of model training.

### 4.3 Feasibility of the data acquisition system in the industrial plant factory

The horizontally mobile data acquisition system in a narrow space is equipped with multiple cameras between a pair of rail frames and terminal rails spaced apart at regular intervals. As it moved in the width direction and along the rail frame in the longitudinal direction, the growth status of the crop was checked by adjusting the position of the camera. The first drive motor for longitudinal movement and the second drive motor for width direction movement were arranged at the same location. This made it possible to safely protect driving parts such as motors in high-temperature and high-humidity growing environments.

The first and second driving units operated in conjunction with each other, longitudinal movement was possible, and the position of the terminal rail could be automatically adjusted through the control box.

The timing belt of the first and second drive units was open type. Owing to its structure, there was no need to separate the first and second drive motors. The beneficial effect of improving the waterproofing effect was achieved by placing it in one waterproof case.

RGB and IR images were captured consistently and automatically in an industrial plant factory. Sometimes, the system instantly stopped when some debris fell into the gap of the rail frame because it caused the slip phenomenon of the motors, causing it to lose balance when moving in parallel, resulting in a stop in motion and not taking images. However, it operated well immediately after the rail frame was cleaned. Despite this issue, consistent images for developing models were adequately and automatically obtained from the system, resulting in good results from the developed models due to the high image quality. As mentioned in the *Materials and methods*, depth images were taken with a RealSense D405 depth sensor on the camera rail of the linear motion guide. However, three weeks later, the images were too corrupted for use in model development. The RealSense D405 depth sensor is assumed to be unsuitable in harsh, high-humidity environments because it operates well when brought back to laboratory conditions but not in the plant factory, even after the condition check. Apart from the depth images, all other images of RGB and IR were taken continuously.

**Figure 10:**
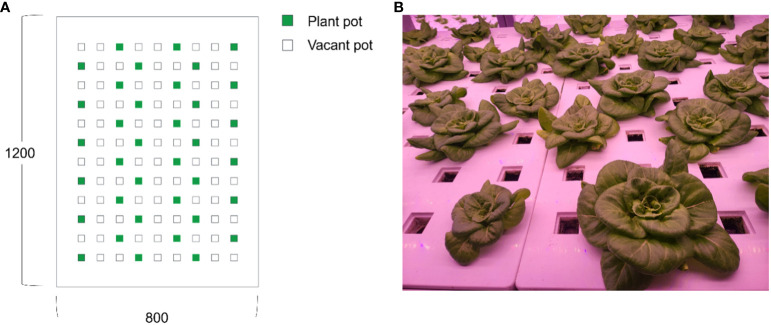
Positions of plant pots in the cultivating rack and grown crops: **(A)** zigzag positions of plants and vacant pots; **(B)** well-grown lettuce without overlapping problems.

In commercial plant factories where the experiment was conducted, crops were planted in zigzag positions and grown at an appropriate density to prevent the overlap of lettuce (**Figure 10**). A total of 36 crops were transplanted and cultivated in a 1,200 mm × 800 mm cultivation rack.

Except for the depth camera, it was feasible to operate in a commercialized plant factory with high humidity and a confined space to obtain data automatically and consistently. It would be more efficient if workers cleaned up the surrounding cultivating beds after touching crops to avoid falling debris in the gap of the aluminum profiles.

### 4.4 Future study

Several attempts have been made to improve this model. For example, it can be improved if the LSTM model is applied to develop time-series models that reflect the exact changes in plant growth on days. Integrating LSTMs can capture the sequential nuances of growth, offer a granular view of development cycles, and enable more targeted interventions. In addition, diverse types of images can be added for model development by collecting them with stable devices that can be utilized under harsh conditions. Enhancing the dataset with thermal or hyperspectral imaging could also unlock new correlations between visible symptoms and the plant’s internal state, leading to more comprehensive growth data. For example, taking depth images with a stereo camera or taking images from the side view can further improve models because they can contain more information, such as the height and volume of plants and the number of leaves, including hidden leaves. These multidimensional data could facilitate a more sophisticated model that predicts weight and assesses plant health and maturity, thereby informing more accurate harvesting times. This information can be used as another variable in the model. Future iterations of the model could also leverage advancements in predictive algorithms to automate the detection of abnormal growth patterns, thereby offering early warnings for potential issues. In addition, the models can be developed into other forms of narrow environments, such as small-scale or home-based systems (Kim et al., 2022), and other varieties with different plant morphogenesis. In the case of diverse species, different morphogenesis can cause different vision-based model performances, with different values of RMSE and R2. Moon et al. performed a growth analysis of plant factory-grown lettuce such as Corbana, Caipira, and Fairy using deep neural networks based on automated feature extraction (Moon et al., 2022a). The result of the model adopting convolutional neural networks was 0.77 of R2. One reason for the low performance can be assumed to be the different morphologies of different crops.

## 5 Conclusion

In this study, we successfully developed a fresh weight prediction model for butterhead lettuce using computer vision that can also be used on-site in plant factories. This application represents the first development of a noninvasive weight estimation system based on automatic data acquisition, especially for commercialized plant factories with narrow, crowded, and high-humidity environments. Specifically, using a linear motion guide, the automatic data acquisition system is adequate for collecting consistent unstructured data onsite. This deployment of the automated system within a plant factory underscores the significant innovation in the data collection method in agricultural technology.

Automatic feature extraction with a convolutional neural network (CNN) based on images showed a high performance with an R2 of 0.95 and RMSE of 8.06 g compared to any other model for the fresh weight estimation of butterhead lettuce. However, MLP_2 can be more appropriate to be adopted on the spot in the industrial plant factory because the inference time per image was approximately nine times faster than CNN, with a slightly lower value of R2 (0.93) and a slightly higher value of RMSE (9.35 g). Therefore, the superior performance of MLP_2 introduces a breakthrough in precision agriculture, particularly in how data-driven models can be seamlessly integrated into operational workflows. The automatic feature extraction-based models using images as input through a multilayer perceptron (MLP) performed better than any other manual feature extraction-based models since the best performance was 0.90 of R2 and 10.63 g of RMSE from the third-degree of polynomial multivariable regression model with parameters of A and P. Therefore, the automatic feature extraction method using unstructured data is the most appropriate model for predicting fresh weight.

As an onsite automatic data acquisition system, the models should be light and available with lower computing power. The practical implementation of such efficient models in a commercial setting, without reliance on high-power computational resources, illustrates the feasibility and applicability of our approach. The model generated in this study uses automatic feature extraction with unstructured data, but it has a simple structure and shows sufficient performance compared to other models in previous studies. Simplifying complex data processing into a robust yet straightforward model that is accessible for on-site use is another innovative aspect of this study. The model can be applied to other fields such as small-scale home-based cultivation systems. In this study, workers involved in indoor farming were able to measure the fresh weight of crops in a non-destructive way and harvest at the appropriate time.

## Data availability statement

The raw data supporting the conclusions of this article will be made available by the authors, without undue reservation.

## Author contributions

J-SK: Conceptualization, Data curation, Formal Analysis, Investigation, Methodology, Project administration, Software, Validation, Visualization, Writing – original draft, Writing – review & editing. SM: Writing – review & editing, Methodology, Software. JP: Methodology, Writing – review & editing. TK: Methodology, Software, Writing – review & editing. SC: Funding acquisition, Resources, Supervision, Writing – review & editing.

## References

[B1] Adenäuer M. Deuss A. Elasri A. Altendorf S. René S. Enciso SRA.. (2023). OECD-FAO Agricultural Outlook 2023–2032. Paris: OECD Publishing. doi: 10.1787/08801ab7-en

[B2] Ben Hassen T. El Bilali H. (2022). Impacts of the Russia-Ukraine war on global food security: towards more sustainable and resilient food systems? Foods 11, 1–17. doi: 10.3390/foods11152301 PMC9368568 35954068

[B3] Boros I. F. Székely G. Balázs L. Csambalik L. Sipos L. (2023). Effects of LED lighting environments on lettuce (Lactuca sativa L.) in PFAL systems – A review. Scientia Hortic. 321, 112351. doi: 10.1016/j.scienta.2023.112351

[B4] Brain R. Perkins D. Ghebremichael L. White M. Goodwin G. Aerts M. (2023). The shrinking land challenge. Cite This: ACS Agric. Sci. Technol. 2023, 152–157. doi: 10.1021/acsagscitech.2c00250

[B5] Chamara N. Islam M. D. Bai G.(. Shi Y. Ge Y. (2022). Ag-IoT for crop and environment monitoring: Past, present, and future. Agric. Syst. 203, 103497. doi: 10.1016/j.agsy.2022.103497

[B6] Chen Y. C. (2017). A tutorial on kernel density estimation and recent advances. Biostatistics Epidemiol. 1, 161–187. doi: 10.1080/24709360.2017.1396742

[B7] Chen W. T. Yeh Y. H. F. Liu T. Y. Lin T. (2016). An automated and continuous plant weight measurement system for plant factory. Front. Plant Sci. 7. doi: 10.3389/fpls.2016.00392 PMC4815294 27066040

[B8] Farcas A. C. Galanakis C. M. Socaciu C. Pop O. L. Tibulca D. Paucean A.. (2020). Food security during the pandemic and the importance of the bioeconomy in the new era. Sustainability 13 (1), 150. doi: 10.3390/su13010150

[B9] Gang M. S. Kim H. J. Kim D. W. (2022a). Estimation of greenhouse lettuce growth indices based on a two-stage CNN using RGB-D images. Sensors 22, 5499. doi: 10.3390/s22155499 35898004 PMC9331482

[B10] Hati A. J. Singh R. R. (2021). Smart indoor farms: leveraging technological advancements to power a sustainable agricultural revolution. AgriEngineering 4), 728–767. doi: 10.3390/agriengineering3040047

[B11] He K. Zhang X. Ren S. Sun J. Deep Residual Learning for Image Recognition. Available online at: http://image-net.org/challenges/LSVRC/2015/.

[B12] Jiang J.s. Kim H. J. Cho W. J. (2018). On-the-go image processing system for spatial mapping of lettuce fresh weight in plant factory. IFAC-PapersOnLine 51, 130–134. doi: 10.1016/j.ifacol.2018.08.075

[B13] Kim J. S. G. Jeong W. Park S. Yang M. (2022). Development of a fuzzy logic-controlled system for home cultivation of sweet basil. Front Plant Sci. 13, 999106. doi: 10.3389/fpls.2022.999106 36340373 PMC9627040

[B14] Kozai T. Niu G. Takagaki M. (2019) Plant factory: an indoor vertical farming system for efficient quality food production. Available online at: https://books.google.com/books?hl=en&lr=&id=z-C7DwAAQBAJ&oi=fnd&pg=PP1&ots=zDtmsLhkfp&sig=4I5QCwwMEzkE-Rp9Se_-GEAb0oM.

[B15] Kozai T. Sasaki S. (2013). Resource use efficiency of closed plant production system with artificial light: Concept, estimation and application to plant factory. Proc. Japan Academy Ser. B 89, 447–461. doi: 10.2183/pjab.89.447 PMC3881955 24334509

[B16] Lee J. W. (2008). MACHINE VISION MONITORING SYSTEM OF LETTUCE GROWTH IN A STATE-OF-THE-ART GREENHOUSE *. Modern Phys. Lett. B 22, 953–958. doi: 10.1142/S0217984908015668

[B17] Lin Z. Fu R. Ren G. Zhong R. Ying Y. Lin T. (2022). Automatic monitoring of lettuce fresh weight by multi-modal fusion based deep learning. Front. Plant Sci. 13. doi: 10.3389/fpls.2022.980581 PMC9458202 36092436

[B18] Lou M. Lu J. Wang L. Jiang H. Zhou M. (2022). Growth parameter acquisition and geometric point cloud completion of lettuce. Front. Plant Sci. 13. doi: 10.3389/fpls.2022.947690 PMC9558259 36247622

[B19] Mitchell C. A. (2022). History of controlled environment horticulture: indoor farming and its key technologies. HortScience 57, 247–256. doi: 10.21273/HORTSCI16159-21

[B20] Mokhtar A. El-Ssawy W. He H. Al-Anasari N. Sammen S. S. Gyasi-Agyei Y.. (2022). Using machine learning models to predict hydroponically grown lettuce yield. Front. Plant Sci. 13. doi: 10.3389/fpls.2022.706042 PMC8928436 35310645

[B21] Moon T. Choi W. J. Jang S. H. Choi D. S. Oh M. M. (2022a). Growth analysis of plant factory-grown lettuce by deep neural networks based on automated feature extraction. Horticulturae 8 (12), 1124. doi: 10.3390/horticulturae8121124

[B22] Moon T. Park J. Son J. E. (2020). Estimation of sweet pepper crop fresh weight with convolutional neural network. Protected Horticulture Plant Factory 29, 381–387. doi: 10.12791/KSBEC.2020.29.4.381

[B23] Mortensen A. K. Bender A. Whelan B. Barbour M. M. Sukkarieh S. Karstoft H.. (2018). Segmentation of lettuce in coloured 3D point clouds for fresh weight estimation. Comput. Electron. Agric. 154, 373–381. doi: 10.1016/j.compag.2018.09.010

[B24] Ojo M. O. Zahid A. (2023). Non-Destructive Biomass Estimation for Hydroponic Lettuce Production. ASABE Paper No. 2300776. St. Joseph, MI.: ASABE. 1–10. doi: 10.13031/aim.202300776

[B25] Ong H. C. Leong C. H. Tai S. H. (2008). A functional approximation comparison between neural networks and polynomial regression. WSEAS Trans. Mathematics 7, 353–363.

[B26] Petropoulou A. S. van Marrewijk B. de Zwart F. Elings A. Bijlaard M. van Daalen T.. (2023). Lettuce production in intelligent greenhouses—3D imaging and computer vision for plant spacing decisions. Sensors 23 (2), 2929. doi: 10.3390/s23062929 36991638 PMC10052086

[B27] Reyes-Yanes A. Martinez P. Ahmad R. (2020). Real-time growth rate and fresh weight estimation for little gem romaine lettuce in aquaponic grow beds. Comput. Electron. Agric. 179, 105827. doi: 10.1016/j.compag.2020.105827

[B28] Tong Y. Oh M. M. Fang W. (2023). Editorial: Advanced technologies for energy saving, plant quality control and mechanization development in plant factory. Front. Plant Sci. 14. doi: 10.3389/fpls.2023.1193158 PMC10235690 37275252

[B29] World Population Prospects - Population Division - United Nations 2022. Available online at: https://population.un.org/wpp/Graphs/.

[B30] Xu D. Chen J. Li B. Ma J. (2023). Improving lettuce fresh weight estimation accuracy through RGB-D fusion. Agronomy 13 (10), 2617. doi: 10.3390/agronomy13102617

[B31] Zhang R. Liu T. Ma J. (2017). Plant factory: A new method for reducing carbon emissions. in AIP Conference Proceedings (AIP Publishing). 1820 1. doi: 10.1063/1.4977288

[B32] Zhang L. Xu Z. Xu D. Ma J. Chen Y. Fu Z. (2020). Growth monitoring of greenhouse lettuce based on a convolutional neural network. Horticulture Res. 7, 124. doi: 10.1038/s41438-020-00345-6 PMC7395764 32821407

[B33] Zhang Q. Zhang X. Wu Y. Li X. (2022). TMSCNet: A three-stage multi-branch self-correcting trait estimation network for RGB and depth images of lettuce. Front. Plant Sci. 13. doi: 10.3389/fpls.2022.982562 PMC9470961 36119576

